# The Current Situation Regarding Long-Acting Insulin Analogues Including Biosimilars Among African, Asian, European, and South American Countries; Findings and Implications for the Future

**DOI:** 10.3389/fpubh.2021.671961

**Published:** 2021-06-24

**Authors:** Brian Godman, Mainul Haque, Trudy Leong, Eleonora Allocati, Santosh Kumar, Salequl Islam, Jaykaran Charan, Farhana Akter, Amanj Kurdi, Carlos Vassalo, Muhammed Abu Bakar, Sagir Abdur Rahim, Nusrat Sultana, Farzana Deeba, M. A. Halim Khan, A. B. M. Muksudul Alam, Iffat Jahan, Zubair Mahmood Kamal, Humaira Hasin, Munzur-E-Murshid, Shamsun Nahar, Monami Haque, Siddhartha Dutta, Jha Pallavi Abhayanand, Rimple Jeet Kaur, Godfrey Mutashambara Rwegerera, Renata Cristina Rezende Macedo do Nascimento, Isabella Piassi Dias Godói, Mohammed Irfan, Adefolarin A. Amu, Patrick Matowa, Joseph Acolatse, Robert Incoom, Israel Abebrese Sefah, Jitendra Acharya, Sylvia Opanga, Lisper Wangeci Njeri, David Kimonge, Hye-Young Kwon, SeungJin Bae, Karen Koh Pek Khuan, Abdullahi Rabiu Abubakar, Ibrahim Haruna Sani, Tanveer Ahmed Khan, Shahzad Hussain, Zikria Saleem, Oliver Ombeva Malande, Thereza Piloya-Were, Rosana Gambogi, Carla Hernandez Ortiz, Luke Alutuli, Aubrey Chichonyi Kalungia, Iris Hoxha, Vanda Marković-Peković, Biljana Tubic, Guenka Petrova, Konstantin Tachkov, Ott Laius, András Harsanyi, András Inotai, Arianit Jakupi, Svens Henkuzens, Kristina Garuoliene, Jolanta Gulbinovič, Magdalene Wladysiuk, Jakub Rutkowski, Ileana Mardare, Jurij Fürst, Stuart McTaggart, Sean MacBride-Stewart, Caridad Pontes, Corinne Zara, Eunice Twumwaa Tagoe, Rita Banzi, Janney Wale, Mihajlo Jakovljevic

**Affiliations:** ^1^Department of Pharmacoepidemiology, Strathclyde Institute of Pharmacy and Biomedical Sciences, University of Strathclyde, Glasgow, United Kingdom; ^2^Division of Public Health Pharmacy and Management, School of Pharmacy, Sefako Makgatho Health Sciences University, Pretoria, South Africa; ^3^School of Pharmaceutical Sciences, Universiti Sains Malaysia, George Town, Malaysia; ^4^Unit of Pharmacology, Faculty of Medicine and Defence Health, Universiti Pertahanan Nasional Malaysia (National Defence University of Malaysia), Kuala Lumpur, Malaysia; ^5^Essential Drugs Programme, South African National Department of Health, Pretoria, South Africa; ^6^Center for Health Regulatory Policies, Istituto di Ricerche Farmacologiche “Mario Negri” IRCCS, Milan, Italy; ^7^Department of Periodontology and Implantology, Karnavati University, Gandhinagar, India; ^8^Department of Microbiology, Jahangirnagar University, Dhaka, Bangladesh; ^9^Department of Pharmacology, All India Institute of Medical Sciences, Jodhpur, India; ^10^Department of Endocrinology, Chittagong Medical College, Chittagong, Bangladesh; ^11^Department of Pharmacology, College of Pharmacy, Hawler Medical University, Erbil, Iraq; ^12^Facultad de Ciencias Médicas, Universidad Nacional del Litoral, Santa Fe, Argentina; ^13^Department of Endocrinology and Metabolism, Chattogram Maa-O-Shishu Hospital Medical College, Chattogram, Bangladesh; ^14^Bangladesh Institute of Research and Rehabilitation in Diabetes, Endocrine and Metabolic Disorders General Hospital, Dhaka, Bangladesh; ^15^Department of Endocrinology and Metabolism, Bangabandhu Sheik Mujib Medical University Hospital, Dhaka, Bangladesh; ^16^Department of Obstetrics and Gynaecology, Bangabandhu Sheik Mujib Medical University, Dhaka, Bangladesh; ^17^Shaheed Suhrawardy Medical College Hospital, Dhaka, Bangladesh; ^18^Department of Physiology, Eastern Medical College, Cumilla, Bangladesh; ^19^National Institute of Mental Health (NIMH), Dhaka, Bangladesh; ^20^Clinical Fellow, Epsom and St Helier University Hospitals NHS Trust, Surrey, United Kingdom; ^21^Women's Integrated Sexual Health (WISH) 2 Access Choice Together Innovate Ownership Now (ACTION) Project, Handicap International, Kurigram, Bangladesh; ^22^Human Resource Department, Square Toiletries Limited, Rupayan Center, Dhaka, Bangladesh; ^23^Department of Medicine, Sir Ketumile Masire Teaching Hospital, Gaborone, Botswana; ^24^Faculty of Medicine, University of Botswana, Gaborone, Botswana; ^25^Department of Pharmacy, Postgraduate Program in Pharmaceutical Sciences (CiPharma), School of Pharmacy, Federal University of Ouro Preto, Ouro Preto, Brazil; ^26^Institute of Health and Biological Studies, Universidade Federal do Sul e Sudeste do Pará, Cidade Universitária, Marabá, Brazil; ^27^Group (CNPq) for Epidemiological, Economic and Pharmacological Studies of Arboviruses (EEPIFARBO), Universidade Federal do Sul e Sudeste do Pará, Marabá, Brazil; ^28^Faculdade de Odontologia, Universidade Federal de Pelotas, Pelotas, Brazil; ^29^Pharmacy Department, Eswatini Medical Christian University, Mbabane, Eswatini; ^30^Cape Coast Teaching Hospital (CCTH), Cape Coast, Ghana; ^31^Pharmacy Department, Keta Municipal Hospital, Ghana Health Service, Keta-Dzelukope, Ghana; ^32^Pharmacy Practise Department of Pharmacy Practise, School of Pharmacy, University of Health and Allied Sciences, Volta Region, Ghana; ^33^Department of Dentistry, SP Medical College, Bikaner, India; ^34^Department of Pharmaceutics and Pharmacy Practise, School of Pharmacy, University of Nairobi, Nairobi, Kenya; ^35^Department of Pharmacy, Kenyatta National Hospital, Nairobi, Kenya; ^36^Division of Biology and Public Health, Mokwon University, Daejeon, South Korea; ^37^College of Pharmacy, Ewha Woman's University, Seoul, South Korea; ^38^Friends' Pharmacy Pekan Sg Besi 42G, Kuala Lumpur, Malaysia; ^39^Department of Pharmacology and Therapeutics, Faculty of Pharmaceutical Sciences, Bayero University, Kano, Nigeria; ^40^Unit of Pharmacology, College of Health Sciences, Yusuf Maitama Sule University (YUMSUK), Kano, Nigeria; ^41^National Institute of Health, Islamabad, Pakistan; ^42^Department of Pharmacy Practise, Faculty of Pharmacy, The University of Lahore, Lahore, Pakistan; ^43^Department of Child Health and Paediatrics, Egerton University, Nakuru, Kenya; ^44^East Africa Centre for Vaccines and Immunisation (ECAVI), Kampala, Uganda; ^45^Paediatric Endocrinologist, School of Medicine, College of Health Sciences, Makerere University, Kampala, Uganda; ^46^National Resources Fund (FNR), Montevideo, Uruguay; ^47^University Teaching Hospital Group, Department of Pharmacy, Lusaka, Zambia; ^48^Department of Pharmacy, School of Health Sciences, University of Zambia, Lusaka, Zambia; ^49^Department of Pharmacy, Faculty of Medicine, University of Medicine, Tirana, Albania; ^50^Department of Social Pharmacy, Faculty of Medicine, University of Banja Luka, Banja Luka, Bosnia and Herzegovina; ^51^Agency for Medicinal Products and Medical Devices of Bosnia and Herzegovina, Banja Luka, Bosnia and Herzegovina; ^52^Department of Medicinal Chemistry, Faculty of Medicine, University of Banja Luka, Banja Luka, Bosnia and Herzegovina; ^53^Department of Social Pharmacy and Pharmacoeconomics, Faculty of Pharmacy, Medical University of Sofia, Sofia, Bulgaria; ^54^State Agency of Medicines, Tartu, Estonia; ^55^Department of Health Policy and Health Economics, Eotvos Lorand University, Budapest, Hungary; ^56^Syreon Research Institute, Budapest, Hungary; ^57^Center of Health Technology Assessment, Semmelweis University, Budapest, Hungary; ^58^Faculty of Pharmacy, UBT Higher Education Institute, Pristina, Kosovo; ^59^Independent Researcher, Riga, Latvia; ^60^Department of Pharmacy, Faculty of Medicine, Institute of Biomedical Sciences, Vilnius University, Vilnius, Lithuania; ^61^Department of Pathology, Forensic Medicine and Pharmacology, Faculty of Medicine, Institute of Biomedical Sciences, Vilnius University, Vilnius, Lithuania; ^62^Chair of Epidemiology and Preventive Medicine Jagiellonian University, Medical College, Kraków, Poland; ^63^HTA Consulting, Kraków, Poland; ^64^Faculty of Medicine, Public Health and Management Department, “Carol Davila” University of Medicine and Pharmacy Bucharest, Bucharest, Romania; ^65^Health Insurance Institute, Ljubljana, Slovenia; ^66^Public Health Scotland, Edinburgh, United Kingdom; ^67^Pharmacy Services, Greater Glasgow and Clyde (NHS GGC), Glasgow, United Kingdom; ^68^Drug Department, Catalan Health Service, Barcelona, Spain; ^69^Department of Pharmacology, Therapeutics and Toxicology, Universitat Autònoma de Barcelona, Barcelona, Spain; ^70^Department of Management Science, Business School, University of Strathclyde, Glasgow, United Kingdom; ^71^Independent Consumer Advocate, Brunswick, VIC, Australia; ^72^Department of Global Health Economics and Policy, University of Kragujevac, Kragujevac, Serbia; ^73^Faculty of Economics, Institute of Comparative Economic Studies, Hosei University Tokyo, Tokyo, Japan

**Keywords:** Africa, biosimilars, cross-national study, drug utilisation, Europe, health policy, insulin glargine, prices

## Abstract

**Background:** Diabetes mellitus rates continue to rise, which coupled with increasing costs of associated complications has appreciably increased global expenditure in recent years. The risk of complications are enhanced by poor glycaemic control including hypoglycaemia. Long-acting insulin analogues were developed to reduce hypoglycaemia and improve adherence. Their considerably higher costs though have impacted their funding and use. Biosimilars can help reduce medicine costs. However, their introduction has been affected by a number of factors. These include the originator company dropping its price as well as promoting patented higher strength 300 IU/ml insulin glargine. There can also be concerns with different devices between the manufacturers.

**Objective:** To assess current utilisation rates for insulins, especially long-acting insulin analogues, and the rationale for patterns seen, across multiple countries to inform strategies to enhance future utilisation of long-acting insulin analogue biosimilars to benefit all key stakeholders.

**Our approach:** Multiple approaches including assessing the utilisation, expenditure and prices of insulins, including biosimilar insulin glargine, across multiple continents and countries.

**Results:** There was considerable variation in the use of long-acting insulin analogues as a percentage of all insulins prescribed and dispensed across countries and continents. This ranged from limited use of long-acting insulin analogues among African countries compared to routine funding and use across Europe in view of their perceived benefits. Increasing use was also seen among Asian countries including Bangladesh and India for similar reasons. However, concerns with costs and value limited their use across Africa, Brazil and Pakistan. There was though limited use of biosimilar insulin glargine 100 IU/ml compared with other recent biosimilars especially among European countries and Korea. This was principally driven by small price differences in reality between the originator and biosimilars coupled with increasing use of the patented 300 IU/ml formulation. A number of activities were identified to enhance future biosimilar use. These included only reimbursing biosimilar long-acting insulin analogues, introducing prescribing targets and increasing competition among manufacturers including stimulating local production.

**Conclusions:** There are concerns with the availability and use of insulin glargine biosimilars despite lower costs. This can be addressed by multiple activities.

## Introduction

Prevalence rates for diabetes mellitus continue to rise across countries and continents (1–7). This has been exacerbated by over 100% increases in incident cases of diabetes mellitus from 1990, rising to 22.936 million in 2017 (8), which has resulted in an estimated 463 million people worldwide with diabetes mellitus in 2019 (8, 9). These prevalence rates are expected to grow unless addressed. The costs of medicines to treat patients with diabetes, coupled with the cost of associated complications, has increased the global economic burden of diabetes, including both direct and indirect costs, from US$1.3 trillion in 2015 to an estimated US$2.1 to US$2.5 trillion by 2030, equating to 2.2% of Gross Domestic Product (GDP), with most costs as indirect costs (10, 11). The high economic burden of diabetes is amplified by diabetes currently among the leading causes of non-traumatic lower-extremity amputation and blindness worldwide, with patients also at greater risk of cardiovascular disease (9). In view of this, patients with diabetes should be carefully managed. This increasingly includes greater use of long-acting insulin analogues, which were developed to reduce the risk of hypoglycaemia, especially nocturnal hypoglycaemia, and provide greater convenience for patients with a reduced number of injections (9, 12–17).

As a result, we are now seeing usage of long-acting insulins exceed human insulins in upper-middle and high-income countries (18–20). This increase, combined with growing prevalence rates, has resulted in the global insulin market valued at US$24 billion in 2018 and envisaged to grow at a compounded rate of 4.9% (8, 21). Expenditure on long-acting insulin glargine is also growing and envisaged to reach up to US$9.26 billion by 2025 (22), assisted by increasing sales of patented 300 IU/ml insulin glargine (Toujeo®—Gla-300). Increasing use of Gla-300 has helped offset losses from the increasing sales of biosimilar 100 IU/ml insulin glargine (22). Global sales of insulin detemir were US$2.7 billion in 2015 and growing at 7.5% per year (23), and annual sales of insulin degludec are also growing. Sales of insulin degludec grew 1.8-fold in 2017 vs. 2016, rising to US$1.11 billion in 2017 (24). This increase has been supported by studies demonstrating the cost-effectiveness of degludec compared with other long-acting insulin analogues (25–28).

However, there are concerns in some countries regarding the additional costs of long-acting insulin analogues vs. Neutral Protamine Hagedorn (NPH) and other insulins, and whether these additional costs represent value (29, 30). These concerns and issues are reflected by long-acting insulin analogues currently not being included in the World Health Organization Essential Medicines List (WHO EML) (31). In addition, long-acting insulin analogues have not been funded in the public healthcare system in Brazil following advice from the National Health Technology Agency (CONITEC) as the considerably higher price could not be justified by only modest additional patient benefits (32, 33). Consequently, patients in Brazil have been required to fund the costs of long-acting insulin analogues themselves unless there is a successful court case (29). Alongside this, there have been calls for disinvestment in insulin glargine (29); however, this is changing (34). The same situation persists in Bangladesh where the Government only funds NPH and other insulins in public hospitals until funds run out, and in parts of India (18, 35).

Similarly, long-acting insulin analogues are currently not funded within the public healthcare systems in South Africa and Zambia due again to concerns with higher costs than basal/ NPH insulins and no perceived clinical advantage (20, 36), with limited funding and use generally of long-acting insulin analogues across Africa (37). This mirrors the findings of Ewen et al. (18), who showed considerable price differences between human insulins (US$5 per 10 mL of 100 IU/mL) and long-acting insulin analogues among a range of lower- and middle- income countries (LMICs) (US$33), which limited their funding and use. The situation is exacerbated across Africa and other LMICs by concerns with access and availability of insulins including NPH and short-acting insulins in the first place as well as limited facilities and equipment to diagnose diabetes and enable patients to regularly monitor their insulin levels to reduce hypoglycaemia (18, 36, 38–41). However, some studies have shown that the higher acquisition costs of long-acting insulin analogues can potentially be offset by savings from averted costs of hypoglycaemia and other diabetes-associated complications (42–45).

Biosimilars are a potential way forward to reduce the costs of long-acting insulin analogues, similar to other situations (46–48), thereby potentially enhancing their reimbursement and funding. As an example, among hospitals in Denmark, expenditure on adalimumab decreased by 82.8% from September 2018 to December 2018 with a switch to adalimumab biosimilars at considerably lower prices (46). In England, the uptake of biosimilars has appreciably increased with multiple activities among health authorities and other key stakeholder groups, which include education and prescribing targets for biosimilars (49). These multiple activities enhancing biosimilar use resulted in estimated savings from infliximab, etanercept and rituximab in England at GB£99.4, GB£60.3, and GB£50.4 million, respectively, in 2017 (48). Expenditure on adalimumab is also envisaged to fall by 75% in England following the availability of biosimilars combined with multiple demand-side measures to encourage their prescribing (48). In the USA, Claytor and Gellad recently calculated that greater use of biosimilars could save up to US$54 billion over the next 10 years (50, 51). Realising these savings though includes addressing any concerns with biosimilars including any misinformation and its impact on any nocebo effect (52).

However, we have seen in practise that potential savings from biosimilar insulin glargine can be limited. In their study, Ewen et al. (18) found among a range of LMICs that median biosimilar prices for insulin glargine in the public sector ranged from only 2–25% lower than the originator depending on the presentation, with sometimes higher prices for biosimilars in private pharmacies. This matches limited price reductions for biosimilar insulin glargine seen in other markets, which are easily matched by the originator company reducing their attractiveness (53). Besides, there can be concerns with switching patients between originators and biosimilars due to different devices and the potential for hypoglycaemia in some countries (53–56); however, this is not universal (35). Concerns regarding the different devices resulted in, for instance, low use of insulin glargine biosimilars (9%) among diabetologists in the UK in 2017 (57). Consequently, some European health authorities have advised against switching between originator and biosimilar insulin glargine despite publications demonstrating similar effectiveness and safety between them (58–61).

We are also aware that the parent company has launched a patented 300 IU/ml formulation of insulin glargine (Gla-300) to further reduce the risk of hypoglycaemia given its longer half-life and more stable bioavailability vs. the 100 IU/ml formulation. In addition, improving patient comfort and convenience (62). Published studies have shown that Gla-300 does improve glycaemic control alongside reducing the incidence of nocturnal hypoglycaemia vs. the 100 IU/ml formulation (25, 62–65). However, some authors and health authorities are not convinced of the benefits of Gla-300 (66), and have restricted its prescribing vs. the 100 IU/ml formulation (67). Overall, these combined factors appear to have reduced the attractiveness of the 100 IU/ml market to biosimilar manufacturers as well as reduced potential savings from the availability of biosimilar insulin glargine 100 IU/ml. However, this is not universal with some commissioning groups in England in December 2018 already achieving utilisation rates of 53.3% for biosimilar insulin glargine vs. total insulin glargine (68). In the US, usage of the biosimilar insulin glargine reached over 40% of the total insulin glargine public market by 2018 (54).

The first priority within a country should be to ensure that NPH insulin and other standard insulins, along with other essential medicines listed in the WHO EML to treat patients with diabetes and associated co-morbidities, are readily accessible and available to all citizens. This is not always the case as seen among several African countries unless there are access programmes (69–72). In addition, there is the necessary equipment and facilities to rapidly diagnosis diabetes, especially given levels of misdiagnosis among a number of LMICs (73, 74), as well as enable patients to routinely monitor their insulin levels at home to prevent complications (37). This is before considering funding more expensive long-acting insulin analogues given the morbidity and mortality associated with diabetes (75). However, the situation can be very different among a number of Asian and European countries.

Consequently, in view of the many issues and challenges identified, we believe there is a need to assess current utilisation and expenditure patterns for long-acting insulin analogues across countries and continents, including biosimilars, and the rationale for the patterns seen. This includes their funding and use in multiple LMICs building on the studies of Ewen et al. (18). Subsequently, investigate the extent of current price reductions and other activities among originator and biosimilar companies to enhance the affordability and use of long-acting insulin analogues. The objective being to suggest potential activities that could be introduced among all key stakeholder groups to enhance future availability and use of biosimilar long-acting insulin analogues across countries to the benefit of all key stakeholder groups. This includes potential strategies to enhance funding and use of lower cost biosimilars, which will likely vary by country and considerations including those countries where affordability is a key consideration.

## Methodology

We adopted a mixed approach to collect utilisation and expenditure data for insulins in general, and long-acting insulin analogues in particular, across a range of countries and continents, and the subsequent rationale for the patterns seen. We focused on insulin glargine among the long-acting insulin analogues as there are biosimilars currently available across countries. There has also been calls for insulin glargine to be disinvested in view of appreciably increased costs vs. NPH and other insulins and limited perceived additional value (29).

The European regions selected included both Western European and Central and Eastern European (CEE) countries as they constitute a range of countries with different economic powers, geographies and populations (76) to robustly compare and contrast the different approaches to long-acting insulin analogues and biosimilar insulin glargine preparations. However, the principal focus was on CEE countries as there has been appreciably lower utilisation of originator biologic medicines among these countries vs. Western European countries given their high costs and associated high patient co-payments (77–79). Consequently, there should be greater potential for long-acting insulin biosimilar analogues among CEE countries. The limited number of Western European countries and regions for comparative purposes included Italy, Spain (Catalonia) and the UK (Scotland). These countries and regions were chosen as they have instigated multiple activities to enhance biosimilar use (54, 80–83).

The data collection approach was opportunistic and adapted depending on the availability and access of pertinent data across countries, as well as the situation within countries. Among the European countries, the principal focus was on reimbursed utilisation and expenditure data. Utilisation and expenditure data was based on health authority and health insurance company data. We have extensively used these databases in the past as they provide robust data sets which are regularly audited (76, 84, 85). Consequently, these databases are seen as a reliable source for comparing utilisation and expenditure data across countries (84). The only exception in Europe was Kosovo where the data was based on imports. This is because formal reimbursement of medicines has not yet started in Kosovo; hopefully, by the end of 2021 or the first quarter of 2022. We have used this approach in Kosovo in previous studies (86, 87). Utilisation data where available was broken down into Defined Daily Doses (DDDs) to aid comparisons between countries. DDDs are a well-recognised measure for comparing utilisation patterns between countries (88, 89). We have used DDDs before in multiple publications when assessing utilisation and expenditure patterns across disease areas and countries (76, 90–95). Expenditure and pricing data typically remained in the local currency across Europe without conversion to either Euros or US$ as the principal focus especially in Europe was on percentage differences in reimbursed prices over time between the originator and the different biosimilars rather than actual price levels. Pricing data has also been collected for a number of African and Asian countries, especially where long-acting insulins are currently not reimbursed, and converted to US$ where pertinent for comparative purposes using current exchange rates (https://www.xe.com/currencyconverter/).

The Asian countries chosen included Bangladesh, India, Korea, Malaysia and Pakistan. These countries again provide a range of countries based on their population size, economic status—-Gross Domestic Product (GDP) per capita (96), the extent of universal healthcare, geography, level of co-payments, as well as financial consequences when family members become ill (97–99).

Pricing and utilisation data were also included from a range of South American and African countries since long-acting insulin analogues are not routinely listed or reimbursed within the public healthcare systems in a number of these countries or recommended in national treatment guidelines (20, 37, 100, 101), although this is changing. Since originator or biosimilar long-acting insulin analogues in these countries are typically dispensed in private community pharmacies or drugs stores, or in private hospitals, feedback from physicians, pharmacists and key personnel working within pharmacies, combined with local knowledge, has been used to provide information on utilisation and prices of the different long-acting insulin analogue preparations together with changes in recent years and any rationale. We have used this approach before when national datasets are not routinely available (97–99, 102). Similar to previous projects, impressions were provided from physicians and pharmacists when no Ministry of Health or other robust data sets were available to document changes in utilisation and prices of insulin glargine as well as other insulin preparations in recent years. That is, if other information sources were unavailable due to issues of confidentiality and local culture since we were not paying physicians or pharmacy personnel for their time (35, 97–99). The data from community pharmacies and drug stores have been supplemented with utilisation and expenditure data from hospitals where available. The hospitals were typically selected to provide a representation of the situation within a country. In Brazil, Korea and Pakistan, data was extracted from the MIDAS-IQVIA International database as well as government sources to provide current data on utilisation and expenditure patterns. In the case of Korea, this builds on a recent study with infliximab biosimilar (103).

Feedback from the senior-level co-authors and some of the community pharmacists involved in the study will be also used to suggest potential ways to enhance future listing, funding and use of long-acting insulin analogues including biosimilars where currently these are not available/not routinely funded and used within public healthcare systems to the benefit of all key stakeholders.

Ethical approval for this study was not required according to national legislation and institutional guidelines in line with previous studies in similar circumstances (97–99, 102, 104, 105). However, where pertinent, all pharmacists freely provided the requested information having been allowed to refuse to participate if wished. This is in line with previous studies undertaken by the co-authors in this and related areas, which include analysis of policies to enhance the rationale use of medicines and biosimilars, pricing policies and issues surrounding generics, which involved direct contact with health authority personnel and other key stakeholders (76, 99, 104, 106–109).

## Results

We will first consolidate the findings from those continents where long-acting insulin analogues are not routinely funded before discussing the situation across Europe. This reflects variable listing and funding of long-acting insulin analogues among the countries in these continents vs. upper-middle- and high-income countries. In this way, seek to develop a logical sequence of activities to enhance future funding and use of long-acting insulin analogues starting from a situation where these are currently not being funded within public healthcare systems.

We also know in Canada that Basaglar® insulin glargine biosimilar accounted for 7.8% of all insulin glargine dispensed between July 2016 and June 2018 among the Provinces in Canada (110). These rates are expected to grow with the instigation of further demand-side measures to enhance biosimilar prescribing within the public health system in Canada (111, 112) guiding other countries.

### General

Table 1 summarises listing and funding of long-acting insulin analogues among the studied countries in Africa, Asia and South America, with long-acting insulin analogues funded in all the studied European countries. Whilst there was typically a greater likelihood of funding of the long-acting insulin analogues among the higher income countries, and a lack of listing among the lower-income countries, this was not universal.

**Table 1:** Current GDP/capita and listing of long-acting insulins in country EMLs/funding among the studied countries across Africa, Asia, and South America.

**Country**	GDP/capita (US$)^*^	**Current funding/listing in EMLs**	
		**Listed in the EML/funded in the public healthcare system**	**Not listed/funded in the public healthcare system**
Uganda	794.3		√
Pakistan	1248.7		√
Zambia	1305.1		√
Kenya	1816.5	√	
Bangladesh	1855.7		√
India	2099.6	√	
Ghana	2202.1	√	
Nigeria	2229.9	√	
Eswatini	3894.7		√
South Africa	6001.4		√^**^
Botswana	7961.3		√
Brazil (national)	8717.2		√^**^
Argentina	9912.3	√	
Malaysia	11,414.2	√	
Uruguay	16,190.1	√	
Korea	31,846.2	√	

* *GDP/capita based on the latest World Bank data (96)*;

** *will change depending on appreciable price reductions*.

### Africa

Generally among the studied African countries, there is limited utilisation of long-acting insulin analogues in the public healthcare system due to cost differences with human insulins and issues of affordability (Table 1). In addition, many African countries currently struggle with early diagnosis of diabetes due to a lack of facilities and personnel with patients also struggling to routinely monitor their insulin levels (37, 41). In view of concerns with costs and affordability, there is variable listing of long-acting insulin analogues within the EML of the studied African countries (Table 1), which limits the potential market for biosimilar insulin analogues in practise (20, 38).

#### African Countries Where Long-Acting Analogues Are Not Funded in the Public Healthcare System

Currently, long-acting insulin analogues (detemir) are registered in Botswana but not currently available within the public healthcare system in Botswana. This is different to short, intermediate-acting and pre-mixed insulins (37). Lowering the prices of long-acting insulin analogues considerably through biosimilars would appreciably enhance their listing in the EML and use within the public health system in Botswana, similar to other African countries.

In Eswatini, only standard insulins including soluble insulins, isophane, and pre-mixed insulins (30/70), are currently available in the public healthcare system (113). In addition, whilst insulin protaphane is not listed in the current EML, it is stocked in some government hospitals and Army clinics to enhance patient care. Insulin glargine though is available within private hospitals in Eswatini. Current wholesale prices for insulin protaphane 5 × 3 ml pensets is ZAR 633.00 (US$42.80), with insulin glargine 100 IU/ml 17% higher at ZAR727 (US$49.16) for a 5 × 3 ml penset (similar DDD). This is encouraging as reductions in the price of insulin glargine toward soluble and isophane insulin, coupled with additional education of key Government personnel, could potentially enhance future EML listing and funding of biosimilar insulin glargine in the public healthcare system in Eswatini.

Insulin utilisation increased to 3.19 billion DDDs in the public health system in South Africa in 2019, an increase of 11.1% compared with 2018, reflecting increased diabetes prevalence rates (3). However, expenditure went down by 3.6% suggestive of the additional savings that can be made through economies of scale as part of procurement practises (Table 2). Currently, long-acting insulin analogues are not listed on the essential medicine list in the public healthcare system in South Africa due to higher costs than basal/NPH insulins and no perceived clinical advantage (20). Cost considerations are critical in South Africa when considering reimbursement and funding of treatments within the public system in view of growing prevalence rates for NCDs (including diabetes) coupled with the ongoing implementation of universal healthcare (3, 37, 114).

**Table 2:** Current contract prices for different insulin preparations among public hospitals in South Africa.

**Insulin type**	**Trade name**	EML status^*^	Price^**^
Intermediate-acting (human) and Intermediate-acting combined with fast-acting	Protaphane® HM, 100 iu/ml, disposable pen (5 × 3 ml) and Actraphane® HM 30/70, 100 iu/ml, disposable pen (5 × 3 ml)	EML	ZAR164.10 (US$11.11)
Glargine, long-acting insulin analogue	Optisulin® 100 iu/ml cartridges (5 × 3 ml); pens provided free of charge	NON-EML	ZAR460.40 (US$31.41)
Glargine, long-acting insulin analogue (originator)	Lantus® 100 iu/ml, vial (1 × 10 ml)	NON-EML	ZAR534.57 (US$36.25)
Detemir, long-acting insulin analogue (originator)	Levemir® 100 iu/ml, disposable pen (5 × 3 ml)	NON-EML	ZAR639.20 (US$43.61)

*NB:*

* *EML. Essential medicine list*;

** *Contract price in South African Rand (ZAR) listed on contract circular RT297–2019 (accessed 7 February 2021 and subsequently converted to US$)—available online at: http://www.treasury.gov.za/divisions/ocpo/ostb/bidders/CMD%2024%20RT297-2019.pdf*.

Despite prices of long-acting insulin analogues falling through increasing competition (Table 2); the current 2.8-fold difference in price between intermediate-acting insulins and the lowest price insulin glargine (DDD basis for 5 pens) is still cost-prohibitive for consideration for EML listing in South Africa. The ministerially appointed South African National Essential Medicines List Committee recently reviewed long-acting insulin analogues; however, they did not recommend their use at tertiary and quaternary level of cares due to continued cost differences (20). Consideration of therapeutic grouping of intermediate-acting and long-acting insulin analogues is ongoing and could assist with pooled procurement/tendering and possibly enabling access to long-acting insulin analogues from primary to quaternary level of care in the future. However, this must be at more affordable prices. Currently there is only a 22.6% price difference between the different insulin glargine preparations (5 by 3 ml pen sets) available on the public sector contract on a DDD basis and considerable price differences still exist between the lowest priced insulin glargine and intermediate-acting insulins (Table 2).

In Uganda, long-acting insulin analogues including insulin glargine are currently not listed in the Ugandan EML (Table 1). This is a concern in view of the high rates of hypoglycaemia currently seen; however, reflects issues of affordability in Uganda, which is a major issue (115). Currently, prices for insulin glargine within the healthcare system in Uganda vary between US$15–$35/pen depending on whether this is a biosimilar or originator, and whether hospital or community pharmacy. Typically, adolescents with diabetes require 2 pens/month, with overall costs considerable higher than US$8–10 for soluble insulin, NPH insulin at $9–10, with premixed insulins at $10–15 all at 1,000 IU (i.e., 10 ml of 100 IU/ml) with each 10 ml vial lasting ~25–30 days. It is believed that prices of biosimilar insulin glargine will need to fall appreciably for listing of long-acting insulin analogues in the National EML and encourage appreciably greater use.

In Zambia, stock-outs of insulins listed in the EML among public facilities are a concern with patients having to principally purchase their insulin from private pharmacies subject to 100% out-of-pocket payments (36). To address this, the Government in the Republic of Zambia has been routinely purchasing insulins listed in the Zambian EML, which includes protaphane as its longer-acting insulin with no long-acting insulin analogues, including biosimilar insulin glargine, currently listed in the Zambian EML due to issues of affordability and value (36, 38). Usage of insulin protaphane has increased within the University Teaching Hospitals in Lusaka in recent years reflecting increasing prevalence rates, with this growth rate likely to continue. It is believed that prices of long-acting insulin analogues, including biosimilar insulin glargine, would need to fall substantially to nearer insulin protaphane on a monthly basis for insulin glargine to be prescribed and funded within the public healthcare system in Zambia.

#### African Countries Where Long-Acting Analogues Are Funded in the Public Healthcare System

Whilst long-acting insulin analogues, insulin glargine and determir, are currently listed in the Ghanaian EML (18, 116), they are currently not mentioned in the Ghanaian Standard Treatment Guidelines (STGs) nor reimbursed within the National Health Insurance Scheme in Ghana (101). This is reflected by increased dispensing of soluble, premixed and isophane insulins (1,000 IU) to 9,872 units in Cape Coast Teaching Hospital in 2019 and 7,468 units by mid 2020, up from 8,883 in 2018, with only very limited use of insulin glargine (3 ml 100 IU/ml) at just 4 packs in early 2020.

A similar increase in the use of premixed 30/70 insulin was seen within Keta Hospital, in Ghana, rising from 580 packs in 2015 to 802 in 2019 (38.3%), with this increase expected to continue. However, currently in Keta Hospital, there is no prescribing of long-acting insulin analogues due to price differences and issues of affordability between the various insulin types. Issues of affordability generally with insulins in Ghana are reflected by Novo Nordisk offering insulin free to children to improve their care under its CDiC initiative in Africa (117). Similar to Botswana and other African countries, prices of long-acting insulin glargine will have to fall considerably via lower cost biosimilars to enhance their availability and usage within the public health system in Ghana.

The management of NCDs, including diabetes, is a growing priority in Kenya with NCDs now accounting for an appreciable number of in-patient beds and growing mortality (118, 119). Key concerns and challenges include a lack of diagnosis, issues of management exacerbated by affordability problems as well as control of symptoms. These issues and concerns have resulted in a number of initiatives in recent years (69, 70, 120). These include the Base of Pyramid (BoP) project, with a ceiling price of KSh 500–600 (US$5-6) for insulin Mixtard® insulin to help address affordability concerns. This equates up to a two-thirds price reduction from the regular price of 1,800 KSh (41, 70). Consequently, there has been very variable availability and use of long-acting insulin analogues in Kenya in recent years. This includes the leading tertiary hospital in Kenya—Kenyatta National Hospital (KNH), a level six hospital (120)—where there has been an increasing use of long-acting insulin analogues in recent years; however from a low base. Utilisation of insulin glargine rose from 0.51% of total insulins (DDD basis) in 2015 to currently 3.4–3.6% of total insulin use (DDD basis). Further use of long-acting insulin analogues is hampered though by considerably higher costs compared with standard insulins. Affordability concerns are also reflected in limited or no use of long-acting insulin analogues, including potentially biosimilars, outside of KNH, with patients struggling to fund even basic insulins despite access programmes (41, 70). Consequently, similar to other African countries, prices of long-acting insulin analogues through biosimilars will need to appreciably fall before there is increased use.

Among three hospitals surveyed in the Northern part of Nigeria, there was also low utilisation of insulin glargine vs. short, medium- and longer-acting human insulins. In 2019, utilisation of insulin glargine ranged from 50 to 100 packs of 5 × 3 ml 100 IU/ml, with prices per pack ranging from N3600 (US$9.47) to N4300 ($11.42). There were similar utilisation patterns in the first half of 2020; however, prices rose from for instance N4000 (US$10.53) to N4500 (US$11.84). This low utilisation of insulin glargine may again reflect cost considerations among physicians and patients, with currently high levels of patient co-payments in Nigeria. This is important as high co-payments can be catastrophic for families in Nigeria when members become ill; however, this is not universal (121, 122).

The low use of insulin glargine in Nigeria was also reflected in a survey involving 11 community pharmacies. The average number of packs of insulin glargine dispensed ranged from 35 to 110 during 2019, with an average of 75, with similar patterns in the first half of 2020. Biosimilar Glaritus® typically only accounted for a small proportion of this at under 10%, potentially reflecting concerns with the quality of non-originator medicines in Nigeria (123) as well as limited price differences in reality between originators and biosimilars, e.g., currently only 4% between the biosimilar and the cheapest originator.

## South America

Insulins in Argentina are currently bought by the various Provinces, e.g., PRODIABA in the province of Buenos Aires, to help with their costs for patients as membership can lead to additional discounts. Biosimilar insulin glargine is available in Argentina; however, there is currently limited price differences with the originator, up to a maximum of 3%, which has impacted on their use in clinical practise.

In their recent study, Dias et al. (32) noted that the utilisation of insulins in Brazil obtained via public tendering increased from 5.61DDDs/1000 inhabitants/day (DIDs) in 2009 to 9.04 DIDs in 2017, with recent figures showing this growth is continuing (124). Overall insulin utilisation rose from 687.51 million DDDs in 2014 to 875.64 million DDDs in 2019 (27.4%), with expenditure also rising 85.1% during this period. These figures did not include long-acting insulin analogues with the National Health Technology Agency (CONITEC) recommending that they should not be reimbursed within the Brazilian NHS due to appreciably higher prices and uncertain benefits vs. NPH and other insulins, with subsequent calls for disinvestment (29). This especially given concerns with rising prevalence rates for diabetes in Brazil, with Brazil already having the fourth highest number of patients with diabetes worldwide (32, 33).

However, these recommendations have recently changed for patients with Type 1 diabetes (T1DM) (32, 100). According to the Ordinance of March 19, 2019, long-acting insulin analogues have now been incorporated into the public healthcare system in Brazil. However, this is conditional on the costs of the analogues being equal to or less than the NPH insulin pens, and prescribed according to the guideline established by the Ministry of Health (125), which builds on current activities in the State of Minas Gerais (34). This has limited their use in practise in the public health system. However, patients can still obtain long-acting insulin analogues via private pharmacies in Brazil but subject to 100% co-payment unless there has been a successful law suit (29, 126).

The maximum consumer prices (PMC) for insulin glargine biosimilar preparations (Basaglar® 100 IU/ml 3 ml single pen refill) in Brazil is currently 51.5% cheaper than the originator. However, this price difference is being eroded with additional discounts from the originator company limiting uptake of the biosimilar in practise. This is a concern as this may dissuade biosimilar manufacturers from launching additional biosimilars in the future.

Uruguay contrasts with Brazil as there has been considerable growth in the utilisation of long-acting insulin analogues in recent years, but from a low base, with this trend continuing. Utilisation of insulin glargine and detemir rose by 345.8% between 2014 and 2019 DDDs in the public system (FNR—Fondo Nacional de Recursos), overall rising from a total of 4,912 DDDs on 2014 to 21,900 DDDs in 2019. However, the share of insulin glargine has gradually decreased from 77.9% of total long-acting insulin analogues in 2014 to 71.6% (June 2020) due to increased prescribing of insulin detemir. Expenditure/DDD for insulin glargine though has remained constant at 51.84 Uruguayan Peso (US$1.21)/DDD over the years with no competition from biosimilars. This may change as the long-acting insulin market becomes more attractive to biosimilar manufacturers, helping all key stakeholder groups with lower prices.

## Asia

There is typically growing use of long-acting insulin analogues among the Asian countries studied except for Pakistan. The increased use has been facilitated by the availability of biosimilars promoted by local companies. This is expected to continue.

### Bangladesh

Among the five surveyed public and private hospitals and institutions in Bangladesh, there was considerable variability in the prescribing of long-acting insulin analogues as a percentage of total insulins (Table 3). These prescribing patterns reflect the fact that among public hospitals in Bangladesh standard insulins such as regular, intermediate and premix insulins are typically provided free-of-charge until monies and supplies run out; however, this is not universal (35). Following this, insulins are generally purchased from pharmacies and drug stores with 100% co-payment. Having said this, there are hardship funds available among some hospitals in Bangladesh to help cover the costs of insulin for the very poor given concerns with the potential catastrophic impact on the family if members have diabetes (35, 127, 128).

**Table 3:** Range of insulin prescribing patterns among the surveyed hospitals in Bangladesh [adapted from (35)].

	**% long-acting insulins vs. other insulins**	**% insulin glargine vs. other total long-acting insulin analogues**	**% insulin glargine among different presentations**	
			**Biosimilar (various)**	**Originator**
Public hospital (Medical university)	10–15%	20–30%	30–40%	60–70%
Public hospital (Medical college)	20–30%	70–80%	70%	30%
Public hospital (Medical college)	45–50%	70–80%	40–50%	50–60%
Private hospital (Medical college)	15–20%	70–90%	98%	2%
Private teaching hospital	20–25%	50–60%	40%	60%

Long-acting insulins are currently subject to 100% co-payment in all circumstances in Bangladesh. Typically, patients with diabetes being treated in private hospitals necessarily purchase their insulins from community pharmacies and drug stores with no purchasing of medicines by the hospital.

High levels of prescribing of long-acting insulin analogues in some of the surveyed hospitals (Table 3) is principally driven by endocrinologists in view of their perceived improved effectiveness as well as patient convenience compared with standard insulins. Standard insulins such as premixed insulins are typically prescribed by non-endocrinologists especially as they are provided free-of-charge until funds run out (35). However, their prescribing is decreasing among this group of physicians in favour of long-acting insulin analogues, with this trend likely to continue.

Affordability can be an issue with the higher priced long-acting insulin analogues in Bangladesh, with Shariful Islam et al. (129) documenting that patients with diabetes paid an average of 35,385 BDT (US$454) per year for their medicines vs. only 1609 BDT (US$21) for those patients without diabetes. Consequently, patients with diabetes and physicians should be cost-conscious; however, this is not always the case with high rates of the prescribing of originator glargine among some hospital physicians despite higher prices (Tables 3, 4). Encouragingly, insulin glargine accounts for an appreciable proportion of the long-acting insulin analogues prescribed among the surveyed hospitals (Table 3) as this was the first long-acting insulin analogue launched (2005), with lower cost biosimilars subsequently launched in 2010 (Table 4). There is though growing prescribing and dispensing of insulin detemir and insulin degludec in recent years.

**Table 4:** Dispensing patterns of different insulin preparations among 76 pharmacies and drug stores in Bangladesh (group one).

	**2019**		**2020**	
**% Dispensed**	**Other insulins (%)**	**Analogues (%)**	**Other insulins (%)**	**Analogues (%)**
0–10%	2.6	11.8	2.6	7.9
21–30%	11.8	18.4	13.2	18.4
31–40%	19/7	15.8	26.3	9.2
41–50%	18.4	15.8	15.8	18.4
51–60%	15.8	19.7	11.8	27.6
61–70%	17.1	14.5	21.1	14.5
71–80%	9.2	2.6	5.3	2.6
81–100%	5.3	1.3	3.9	1.3

*NB: % dispensed is the % of the different insulin preparations dispensed that year. Analogues are principally long-acting insulin analogues including originators and biosimilars. 2020 is up to early December 2020. Figures represent %s dispensed*.

Overall, 167 pharmacies from across Bangladesh in four groups provided data on dispensing and pricing patterns, giving a response rate of 66.8%. This wide range of prescribing patterns for the different insulins (Table 3) is reflected in the dispensing patterns among 76 out of 82 pharmacies contacted (first group of pharmacies) (Table 4). Overall, insulin analogues were the principal insulin dispensed in over 40% of the community pharmacies surveyed in 2020, which was principally insulin glargine. The biosimilars were the principal insulin glargine dispensed in over 50% of these pharmacies surveyed, reaching over 80% in 13% of those surveyed. However, again considerable variation depending on a number of issues including affordability and trust.

Table 5 contains the consolidate data on the prices seen for a range of insulin glargine preparations among the 167 pharmacies and drug stores surveyed. Overall, price rises were seen in a minority of pharmacies (10.8%) in 2020 vs. 2019, with the greatest price increases seen for the biosimilars (11.3 vs. 10.3%). Typically though, there were no change in the prices of the different insulin glargine preparations in the majority of pharmacies and drug stores surveyed (79.3%), with reductions seen 9.8% of surveyed pharmacies and drug stores, greatest for the originator (13.8%).

**Table 5:** Typical selling prices for a range of insulin glargine preparations among 58 pharmacies and drug stores in Bangladesh.

**Manufacturer**	**Packs (100 IU/ml insulin glargine)**	**Typical selling price in pharmacies and drug stores (local currency)**
Lantus® (Sanofi-Aventis)	100 IU/ml 3 ml Pen, 5 × 3 ml pen	1220 BDT (US$14.40), 6100 BDT (US$72.00)
Abasaglar®	100 IU/ml−3 ml Pen; 100 IU/ml – 5 × 3 ml cartridges	1085 BDT (US$12.80) to 3617 BDT (US$42.68)
Glarine®	100 IU/ml—Pen, 5 × 3 ml pens	950 BDT (US$11.21), 4750 BDT (US$56.05)
Larsulin®	100 IU/ml−3 ml vial and pen cartridge	600 BDT (US$7.08)
Vibrent®	100 IU/ml−3 ml vial and pen set	600 BDT (US$7.08)

Taken together, the findings from the different hospital personnel and the wide range of pharmacies and drugs stores surveyed suggests that the usage of long-acting insulin analogues is increasing in Bangladesh, which is principally insulin glargine, reflecting their perceived patient benefits. This is increasingly the biosimilar reflecting issues of affordability.

Biosimilars were also the principal preparations of insulin glargine dispensed in a further set of 15 pharmacies, which accounted for just under 90% of all insulin glargine dispensed (89.4% in 2019 and 88.9% in 2020). There was a 16.7% increase in the number of insulin glargine packs dispensed in these 15 pharmacies between 2019 and 2020.

### India

Biosimilar insulin glargine Glaritus® is typically the only insulin glargine 100 IU/ml dispensed among five purposely selected government hospitals in India. There has been an increase in the prescribing of insulin glargine among these five hospitals from 2014 to 2019 (Table 6); however, falling in 2020 with procurement changes.

**Table 6:** Total annual utilisation of insulin glargine (DDDs) among 5 Government hospitals in India.

	**2014**	**2016**	**2018**	**2019**
Hospital 1	7290.0	8280.0	9022.5	12195.0
Hospital 2	31522.5	34942.5	47587.5	76657.5
Hospital 3	26055.0	29137.5	33232.5	40995.0
Hospital 4	43200.0	49972.5	55125.0	82552.5
Hospital 5	2700.0	3037.5	3937.5	6975.0

This is different to the findings of Ewen et al. (18) who found no insulin, including human insulin, was available in their surveyed provincial and district public hospitals in India and only short-acting insulin was in stock in the one teaching hospital in the state capital. The increasing use of long-acting insulin analogues (insulin glargine) may reflect a growing awareness of the patient benefits with long-acting insulin analogues.

Prices (expenditure/DDD) were generally stable in the surveyed Government Hospitals over the years for insulin glargine at 60 INR (US$0.83)/DDD between 2014 to 2016, rising to 70.0 INR (US$0.97)/DDD in 2017 before falling to 50.93 INR (US$0.70)/DDD. Procured prices in 2020 were 61.60 INR (US$0.85)/DDD.

However, we did see differences in the prices of the different insulin glargine preparations in India in 2020 among 207 community pharmacies surveyed, with prices for Glaritus® glargine at similar prices to the Government hospitals at 382 INR (US$5.28)/3 mls (100 IU/ml). Pharmacy prices ranged from 382 to 650 INR/3 mls (US$ 5.28–8.99) over the years, with 382INR/3 mls the most consistent. This compares to 722 INR (US$9.98)/3 mls (100 IU/ml) as the most consistent price for the originator. Whilst there have also been changes in the prices for both the originator and biosimilars between 2016 and 2020 among the community pharmacies surveyed, with a maximum of 20% in any 1 year, appreciable price differences still exist between the originator and the biosimilars. This price difference may have facilitated the prescribing of biosimilar insulin glargine within the surveyed hospitals in India in recent years where typically insulins are provided free of charge until funds run out as part of moves toward universal healthcare (18, 130, 131).

### Korea, Malaysia and Pakistan

There are currently limited differences in the public price of insulin glargine 100 IU/ml between the originator and biosimilars in Korea reflecting the situation with other biosimilars (103). Price differences between 2017 and 2019 ranged from a price reduction of 0.27% up to a maximum 5.0% difference between the originator and the biosimilar at US$9.04 vs. US$8.59 and US$9.02 per unit depending on the preparation prescribed (132). These limited price differences, coupled with limited demand-side measures generally in Korea to influence physician prescribing including biosimilars (103, 133), resulted in only limited utilisation of biosimilar insulin glargine in Korea (100 IU/ml) in recent years. Usage is growing though from a low of 0.95% of total insulin glargine preparations in 2017 to 4.7% in 2019, with further growth expected.

Within the university hospitals in Malaysia, there is considerable prescribing of long-acting insulin analogues at between 50 and 70% of all insulins dispensed, which is typically higher than seen among a range of hospitals in Bangladesh (35). Similar to India, biosimilars account for up to 90% of all long-acting insulin analogues dispensed in government hospitals. This appreciable utilisation of biosimilar insulin glargine among public hospitals in Malaysia is facilitated by procurement practises which are based on International Non-Proprietary Names (INN), the government's preferentially purchasing of generics and biosimilars from Malaysian companies where possible (134), and the increasing production of biosimilar insulin glargine in Malaysia manufactured to a high standard (135–137). This potentially serves as a model to other countries seeking to enhance the use of biosimilar insulins in their country.

Since insulins are available free of charge among public hospitals in Malaysia, there is currently limited dispensing of biosimilars among the twelve community pharmacies surveyed (Box 1). Most pharmacies typically dispense the originator for patients wishing to purchase insulin glargine as they are generally willing to cover the full costs themselves (Box 1).

Box 1 **Current situation regarding insulin glargine (100 IU/ml) among twelve community pharmacies in Malaysia.**

- Originator
  ⚬ Available in 9 out of the 12 pharmacies surveyed
  ⚬ Sales price ranged from 55.00–75.00 Malaysian Ringgits (US$13.61–18.55)/dispensed preparation
  ⚬ No change in the selling price 2020 vs. 2019 in 5/9 pharmacies−0–20% higher in one, 0–20% lower in three
- Biosimilar
  ⚬ Not sold or unavailable in 11 out 12 pharmacies in 2019 and 10 out of 12 pharmacies in 2020
  ⚬ When sold, the sales price ranged from 50–60 (US$12.37–14.84) Malaysian Ringgits/dispensed preparation
  ⚬ Where sold—typically only a minority of biosimilar insulin glargine dispensed (below 40% of all insulin glargine preparations)

In Pakistan in recent years, there has also been an increase in insulin utilisation reflecting increased prevalence rates for diabetes mellitus (124, 138). There was a 69.5% increase in the total utilisation of insulin in Pakistan from 8.251 million units in 2014 to 13.988 million units in 2019 and rising. This translated into a 152.1% increase in expenditure during this period, enhanced by increasing expenditure on long-acting insulin analogues including insulin glargine which accounted for 9.6% of total expenditure in 2020 despite only accounting for 1.97% of total utilisation. This low utilisation of insulin glargine as a percentage of total insulin in Pakistan vs. Bangladesh and India could be due to a number of issues and activities. These include affordability and co-payment issues for insulins generally in Pakistan, and for long-acting insulin analogues in particular, despite good availability of the different insulins in the country combined with a lack of listing of the long-acting insulin analogues within the national EML (97, 139–141).

Currently the biosimilar (Basagine®) is 20.5% cheaper than the originator among community pharmacies in Pakistan similar to the findings of Ewen et al. (18), i.e. originator (3/10 ml)–−1,132 to 3,858 PKR (US$7.14–24.32), and the biosimilar (3 ml)–−900 PKR (US$5.67). Despite these price differences, and issues of affordability, it appears that the originator (typically 10 ml vials 100 IU/ml) is typically dispensed among the eight community pharmacies surveyed, although there has been an increase in the dispensing of biosimilar Basagine® insulin glargine in recent months with shortages of the originator.

This limited utilisation of the biosimilars in Pakistan vs. Bangladesh, India and Malaysia may reflect greater physician and patient confidence in the originator due to general concerns with the quality of generics in Pakistan (142) coupled with limited price differences in some pharmacies. In addition, only one biosimilar appears to be available among the community pharmacies surveyed in Pakistan vs. a greater range of biosimilars in Bangladesh and India. As a result, no real competition currently among biosimilar manufacturers and the subsequent implications for lower prices.

## Europe

**Figure 1:**
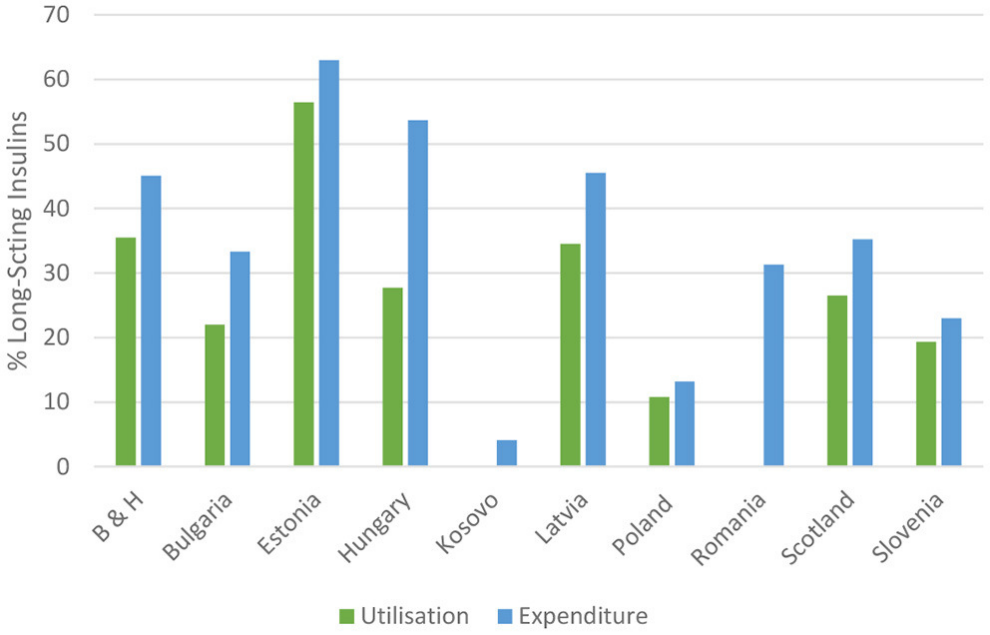
Current utilisation and expenditure on long-acting insulins as a percentage of total insulins among European countries in either 2019 or 2020. NB: B & H, Bosnia and Herzegovina; DDD based with differences in expenditures based on local currencies.

There has typically been growing utilisation and expenditure on long-acting insulin analogues among European countries in recent years. This is reflected in Figure 1 where the latest utilisation of long-acting insulins as a percentage of total insulins is documented. The high documented rates among European countries including Bosnia and Herzegovina, Estonia, Latvia and Romania, reflects increased promotion and other activities by the originator companies of long-acting insulin analogues. In addition, endorsement of the value of long-acting insulin analogues among all key stakeholder groups including the health authorities, physicians and patients. This endorses comments by Ewen et al. that the prescribing of long-acting insulin analogues has been growing among upper-middle and high-income countries in recent years (18, 143).

The greatest increase in the utilisation of long-acting insulin analogues as a percentage of total insulins (based on DDDs) was seen in Poland (210.6%) among the studied countries but from a low base, with the least change in Slovenia (10.7%) but from a higher base.

There has also been considerable variation in the use of biosimilar insulin glargine vs. total insulin glargine across Europe (Table 7). The contrasting rates across Europe reflect a number of key issues and activities. These include the extent of prescribing of Gla-300 in practise, which reached a maximum of 58.0% of total insulin glargine (DDD basis) in Hungary, initial as well as final differences in the prices of originator vs. biosimilar insulin glargine (100 IU/ml), price reductions by the originator company over time (Table 7) as well as the extent, or lack of them, of ongoing demand-side measures to enhance the preferential prescribing of biosimilar insulin glargine 100 IU/ml vs. the originator within individual European countries.

**Table 7:** Key characteristics of the insulin glargine market (100 IU/ml) among European countries.

	**Bio Usage (%)**	**Price difference at launch (Bio vs. Originator) %**	**Price difference originator over time (%)**	**Latest price difference (Bio vs. Originator—%)**	**% Gla-300 (latest)**
Albania	NA	NA	−32.2	NA	45.3
Bosnia and Herezegovina	6.2	0.0	−11.3	−7.9	52.1
Bulgaria	11.0	−4.7	−10.8	−5.7	
Catalonia	12.4	−23.1	−23.1	Similar	28.1
Estonia	0.69	−16.4	−24.9		55.4
Hungary	24.6	−28.2	−21.1	−1.6	58.0
Italy	25.0		−52.3	−24.0	30.4
Latvia	NA	NA	−14.4	NA	51.4
Lithuania	26.5	−12.3	−21.1	0.0	39.0
Poland	44.8	−24.6	−31.1	−0.2	37.1
Scotland	19.5	−18.1	−9.0	−7.5	9.3
Slovenia	15.7	−22.9	−20.3	−9.9	

*NB: Bio usage, % insulin glargine biosimilar vs. total insulin glargine 100 IU/ml on a DDD basis; Orig, Originator; Gla-300, TOUJEO 300 IU/ml on a DDD basis; Blanks represent no data available; NA, Biosimilars not yet launched; Latest price difference either 2019 or 2020 depending on available data; price differences calculated on a DDD basis using local currencies*.

Overall, there was growing utilisation of biosimilar insulin glargine in Italy, Lithuania, Hungary, Poland and Scotland over time, greatest in Poland. In Poland, a flat reimbursement rate with patients paying the price difference for a more expensive originator may explain their high use of biosimilar insulin glargine (144, 145). Alongside this, the Ministry of Health and others in Poland are seeking to grow the prescribing of biosimilars to save resources, with Poland a leading producer of biosimilars in Europe (144, 146). However, overall utilisation of the 100 IU/ml biosimilar as a percentage of total insulin glargine has been reduced by the promotion of Gla-300 with this reaching 37.1% of total insulin glargine by early 2020 aided by a price reduction of 5.8% between 2017 and 2020.

In Scotland, there has been limited utilisation of Gla-300 following prescribing restrictions, mirroring the impact of prescribing restrictions and other activities in Scotland which have been introduced to limit the prescribing of medicines where there are concerns with their role and value (67, 147–149).

The situation in Italy, Lithuania, Hungary, and Poland contrasts with other European countries including Albania, Kosovo, and Latvia, where biosimilar insulin glargine has not yet been launched as well as Estonia and Romania where there is currently very limited use. However, the utilisation of biosimilar 100 IU/ml in Italy, Lithuania, and Poland is moderated by appreciable usage of Gla-300 (Table 7).

Reasons for current unavailability of biosimilar insulin glargine in Kosovo include concerns with their effectiveness and safety vs. the originator. Reasons for no availability or limited use of biosimilar insulin glargine in Albania, Estonia and Latvia include limited attractiveness of the market following multiple activities by the originator company. These include the originator company continually promoting both the originator 100 IU/ml formulation as well as Gla-300 alongside reducing the prices of its 100 IU/ml formulation to limit any price differences (Table 7). The limited uptake of biosimilar insulin glargine in Romania has been influenced by a number of factors including ongoing pricing and reimbursement policies coupled with limited physician incentives or constraints to preferentially prescribing biosimilars. In addition, biosimilar companies may be discouraged from launching their biosimilars in Romania due to current international reference pricing within the country and fears of parallel exportation.

The price reductions for insulin glargine biosimilars when first launched vs. originator prices (Table 7) is similar to the differences seen among LMICs in the study by Ewen et al. (18).

## Suggestions for the Future to Increase the Utilisation of Insulin Glargine Biosimilars

The first step to enhance the attractiveness of the market for manufacturers of biosimilar long-acting insulin analogues is to reimburse them in the first place. Box 2 summarises a number of activities that Governments and others in LMICs can undertake to improve the availability and use of long-acting insulin analogues principally via biosimilars.

Box 2 **Potential activities to enhance funding and availability of lower cost insulin glargine preparations within public healthcare systems.**

- Increased competition can potentially lower the price of insulin glargine through biosimilars, similar to the situation in Bangladesh as well as for other biosimilars (35, 46, 48). This can potentially be achieved by:
  ⚬ By Governments/procurement agencies building on the WHO prequalification initiative to enhance imports of biosimilars from other LMICs to help break the current monopoly among three principal manufacturers of insulins (150–152)
  ⚬ Governments/procurement agencies could potentially work with insulin manufacturers and others to make long-acting insulin analogues available through biosimilars (or originators) at lower prices. This builds on initiatives in Kenya, Nigeria, and Tanzania (70–72, 117)
  ⚬ Governments (especially across regions) across Africa and potentially Brazil could produce biosimilar insulin glargine within their countries/Regions. For instance, Biocon already supplies insulin analogues to a number of African countries, and has instigated manufacturing capabilities in Malaysia (135, 153). Consortia such as the East African Community consortia, has already advocated increased local production of medicines to address future shortages (154, 155), Aspen in South Africa is currently producing vaccines for COVID-19 under licence (156), and Fiocruz in Brazil is already producing recombinant insulins (157)
- Ideally, biosimilar long-acting insulin analogues should be no more than 30–50% above current prices of NPH and other standard insulins on a daily basis, enhanced by low production costs (158)
- Governments should only list biosimilar insulins (of proven quality) on EMLs where these exist and not originators to further enhance competition among biosimilar manufacturers to help drive down prices
- Parallel to this, Ministries of Health and/or physician groups should heighten local knowledge of the potential patient benefits with long-acting insulin analogues if not already seen through clinical trials and other studies including real-world evidence studies as most comparisons between long-acting insulin analogues and NPH/other standard insulins have taken place in high-income countries

Once low cost biosimilar long-acting insulin analogues are routinely available, funded, and used within countries, including listing on country EMLs and addressing issues of affordability including co-payments, there are a number of educational and other initiatives that can subsequently be undertaken to enhance their use.

Encouragingly, we are seeing growing use of long-acting insulin analogues across Europe (Figure 1) as well as other countries including Bangladesh, India, Malaysia and Uruguay. This would suggest increasing endorsement of their value, enhanced by price reductions. In addition, additions to EMLs as seen in Ghana.

Once available and reimbursed, the situation with biosimilar insulin glargine in Europe appears very different to that for biosimilars for immunological diseases and cancer. For instance by 2017, infliximab and etanercept biosimilars had already accounted for 79 and 54% of the UK market share, respectively (159, 160) and growing. In Scotland, etanercept and infliximab biosimilars had already reached 84 and 94% of total utilisation of these biologics by December 2017, and by December 2019, biosimilars trastuzumab had already accounted for 92% of all trastuzumab and biosimilar adalimumab 87% of all adalimumab (54, 83, 161, 162). We believe the limited use of biosimilar insulin glargine among the European countries surveyed apart from Poland (Table 7) is due to a number of factors. These include promotion of Gla-300 alongside price reductions of the 100 IU/ml formulation by the originator company coupled with limited demand-side measures among European health authorities promoting biosimilar insulin glargine. In addition, there are concerns in some countries that the different devices between originator and biosimilar insulin glargine preparations may adversely impact on rates of hypoglycaemia unless patients are educated on the different devices.

Box 3 builds on the findings to date, coupled with input from the senior-level co-authors, to suggest potential measures that could be instigated among all key stakeholder groups to enhance the future use of biosimilar insulin glargine in the public healthcare systems across countries once long-acting insulins are routinely reimbursed and funded. This is particularly important since encouraging greater use of biosimilar insulin glargine should lead to lower prices, similar to the situation with biosimilars of adalimumab and oral generics (46, 48, 179, 180), as well as encourage companies to produce biosimilars for still patented long-acting insulin analogues. This builds on the WHO prequalification initiatives (152, 181), and is seen as critical given the envisaged increase in prevalence rates and costs of diabetes, and the need to appropriately manage patients requiring insulin to reduce hypoglycaemia and improve adherence rates to reduce future complications.

Box 3 **Potential activities to enhance the prescribing and dispensing of biosimilar insulin glargine within public healthcare systems.**

**Educational initiatives**

- Educate all key stakeholder groups where pertinent regarding similar effectiveness and safety between originator and biosimilar long-acting insulin analogues. This includes actively disseminating the findings from current and future studies to avoid/reduce any nocebo effect (52)
- Instigate/help with additional research regarding the potential savings/cost-effectiveness from biosimilar insulin glargine vs. other long-acting insulins as well as NPH and other insulins especially in LMICs—building on current studies. Lower cost biosimilars can potentially enhance access/availability/usage of long-acting insulin analogues in suitable patients where there are concerns—especially given rising rates of diabetes across countries and issues of co-payments/affordability in a number of countries
- Work with patients to ensure they are familiar with the different pens/devices where this is a concern in case of switching between different insulin glargine preparations (and other long-acting insulin biosimilars when they become available) to minimise the potential for hypoglycaemia. This can include an increasing role for nurse specialists and pharmacists - building on their role with patient education in non-communicable diseases including diabetes (163–166)
- Work with patient organisations to reduce any misinformation about biosimilars for long-acting insulin analogues to facilitate greater use—especially where resources/co-payments are an issue. This includes warning patients that the devices may be different between originators and biosimilars where pertinent as this may not always be an issue (35). Alongside this, stressing the importance of good injection techniques (167)

**Other activities**

- Encourage lower prices from companies to stimulate the use of biosimilar insulin glargine (100 IU/ml)—with such activities potentially necessary to (i) address financial concerns with educating patients about the different devices and (ii) reverse current trends in the preferential prescribing of 300 IU/ml insulin glargine (TOUJEO) vs. biosimilar insulin glargine (100 IU/ml). This could be achieved by:
  ⚬ Introducing annual procurement practices for insulin glargine 100 IU/ml— with preference given to biosimilar companies coupled with appropriate demand-side measures to enhance their utilisation
  ⚬ Seek to only list biosimilar insulin glargine (100 IU/ml) on national formulary lists/essential medicine lists when prices are favourable where currently this does not happen, with the potential for delisting insulin glargine from originator companies as familiarity with the biosimilars grows—this builds on activities with renin-angiotensin inhibitors in Denmark (168)
  ⚬ Expanding tendering/procurement activities for 100 IU/ml insulin glargine including biosimilars as seen for instance in Denmark and Norway with the anti-TNFs for patients with immunological conditions (46, 47). This could be via tendering groups as seen across Europe with the formation of groups across countries, e.g., Beneluxa group, as well as in South America with new medicines for hepatitis C (169–172). As part of this, seek to tender with different companies to encourage greater competition in the future as seen with adalimumab in the UK (48). Greater discounts for insulin glargine biosimilar will enhance formulary listing/reimbursement where there are still concerns with the current role and value of long-acting insulin analogues vs. NPH and other insulins as seen in the current WHO EML
  ⚬ Introduce target prescribing goals (quality indicators) for starting patients on 100 IU/ml biosimilar insulin glargine where possible when prescribing a long-acting insulin analogue. In addition, seek to introduce switching targets provided suitable educational support and funding is in place to address concerns when patients are switched between different devices alongside other demand-side measures to enhance preferential prescribing of biosimilars including financial incentives (80, 108, 173). This recognizes that any quality target introduced by health authorities must be acceptable to key stakeholder groups and measurable (174, 175). Concomitant with this, seek ways to limit the prescribing of 300 IU/ml insulin glargine whilst still patented through prescribing restrictions and other activities - building on successful activities in Scotland (Table 7). European health authorities have been successful with instigating prescribing restrictions in the past (176–178)
- Expanding the remit of the Medicines Patent Pool as well as use of the flexibilities enshrined in the WHO TRIPS agreement to increase access and availability of insulin glargine at affordable prices to reduce current barriers where these exist
- Alongside this, make sure that there is agreement between recommendations for long-acting insulin analogues in National EMLs and STGs—addressing concerns seen for instance in Ghana

## Discussion

We believe this is the first study across multiple countries to assess current utilisation patterns for long-acting insulin analogues including biosimilars and the rationale for the patterns seen.

Whilst encouragingly we are seeing growing utilisation of long-acting insulin analogues in a number of LMICs including Bangladesh, India and Malaysia (section Asia) (35), reflecting their increasing role and value in managing patients with diabetes, this is not universal (Table 1). The situation is changing though in a number of these countries with increasing recognition of the value of long-acting insulin analogues, which will be helped by further price reductions of biosimilar insulin glargine 100 IU/ml. There are a number of activities that Governments and Pan-country consortia can undertake to enhance the availability of low cost insulin glargine including increased local production (Box 2). This builds on ongoing activities with insulin manufacture in Brazil and Malaysia as well as vaccines in South Africa (135, 137, 156, 157). This also builds on the WHO prequalification initiative to stimulate competition among potential biosimilar manufacturers to break the current monopoly among three principal manufacturers of insulins (150–152). We have seen that increased competition has helped to lower the prices of biosimilars, building on similar activities with oral generic medicines (35, 46, 180). As a result, enhance the possibility that all patients with diabetes worldwide that need insulin to control their diabetes have access to the same range of insulin preparations to improve the management of their condition.

The situation is different for higher income LMICs (Table 1) and Europe where long-acting insulin analogues are routinely funded within the healthcare systems. In this situation, we have shown that the originator company has instigated a number of activities to reduce the impact of biosimilars. These include switching promotional efforts toward the 300 IU/ml formulation, which reached as high as 58.0% of total insulin glargine on a DDD basis in Hungary (Table 7). This combined with price reductions instigated by the originator limited the availability and prescribing of biosimilar insulin glargine in practise in a number of these countries (Figure 1, Table 7). In fact, no or limited use of biosimilars to date in Albania, Estonia, Kosovo, Latvia and Romania arising from these activities, exacerbated in Romania by concerns with international reference pricing and issues of parallel exportation. The limited use of biosimilar insulin glargine in Korea is a direct consequence of limited price reductions vs. the originator coupled with limited demand-side measures enhancing their prescribing, similar to other biosimilars in Korea (103). Consequently, there is a need to consolidate activities to address key concerns with biosimilars where these exist including addressing any nocebo effect (Box 3) (52), which build on examples in Poland, Lithuania and Hungary in Europe as well as Bangladesh, India and Malaysia in Asia. This is important as we need to encourage biosimilar manufacturers to invest efforts into developing biosimilars for other long-acting insulin analogues when their patents are lost given envisaged increase in insulin sales over the coming years. Otherwise, the insulin market will continue to be dominated by the same three manufacturers limiting potential price reductions in the future (150, 151).

Box 3 also discusses additional activities that key stakeholder groups could undertake to enhance utilisation and funding for biosimilar insulin glargine 100 IU/ml vs. other long-acting insulin analogues including the patent protected 300 IU/ml formulation of insulin glargine. This builds on experiences across countries with other biosimilars as well as other situations and disease areas. As a result, potentially encouraging more 100 IU/ml formulations to be launched and/ or produced locally, thereby leading to lower prices in the future. This though has to be accompanied by educational and other activities where there are concerns to ensure all key stakeholders are comfortable with the increasing use of long-acting insulin analogue biosimilars. Demand-side measures could include the development of pertinent quality indicators among physicians to preferentially encourage the prescribing of biosimilars as well as education among patients with the help of patient organisations and healthcare professionals including pharmacists (Box 3).

Future studies should concentrate on the costs and outcomes of low cost biosimilars vs. NPH and other standard insulins among LMICs as there has been a paucity of data to date. In addition, the impact of demand-side measures to enhance the prescribing of biosimilar insulin glargine 100 IU/ml where there have been concerns as well as monitoring the impact of ongoing demand-side measures to enhance the preferential prescribing of insulin glargine 100 IU/ml vs. other glargine formulations and other long-acting insulin analogues.

We are aware of a number of limitations with this study. These include the fact that we relied on impressions of changes in the utilisation and prices of different insulin preparations including different insulin glargine preparations in a number of African and Asian countries for the reasons given. We were also not able to gain robust utilisation and expenditure data in all countries apart from European countries and South Africa. We are also aware that there can be differences in comparative utilisation rates of 100 vs. 300 IU/ml insulin glargine that is not reflected in their DDDs (66). However, despite these limitations, we believe our findings are informative and provide pertinent directions for the future.

In conclusion, the increasing use of low-cost biosimilars is essential for countries to sustain healthcare systems given the expected growth in expenditure on medicines to address unmet need including the provision of new premium-priced medicines. The availability of insulin glargine at low prices through a variety of activities, which includes stimulating local production of biosimilars and enhancing competition between manufacturers, should enhance their use and help to fund increasing use of insulins to meet rising demand. The increasing availability and use of biosimilar long-acting insulin analogues will also further generate confidence in their prescribing thereby enhancing future availability, funding and prescribing, which is essential given ongoing resource constraints. However, this will typically require multiple demand-side measures to stimulate their use where there are currently challenges.

This is because there are concerns and issues with biosimilar insulin glargine where available and funded vs. a number of the other biosimilars. Consequently, a number of specific demand-side measures may be necessary to stimulate the prescribing of biosimilar insulin glargine. This includes potentially instigating prescribing restrictions for originator as well as higher strength (300 IU/ml) insulin glargine along with preferential listing 100 IU/ml biosimilar insulin glargine on formularies and EMLs. Such activities could potentially overcome activities by the originator company both in terms of reducing prices to compete with biosimilars as well as switching promotional activities to enhance the prescribing of still patented formulations. There are also concerns with different devices in some markets, although this is not universal. Improved patient education, familiarity and lower prices for biosimilars, coupled with introducing a number of demand-side measures including quality indicators and prescribing restrictions where pertinent and feasible, will enhance their reimbursement, funding and utilisation in the future. We will continue to monitor this.

## Data Availability Statement

The original contributions presented in the study are included in the article/supplementary material, further inquiries can be directed to the corresponding author/s.

## Author Contributions

BG, EA, and RB developed the concept of this paper. All authors subsequently contributed with data from their countries where pertinent as well as developed, critiqued successive drafts, and approved the final version.

## Conflict of Interest

MW and JR work for HTA Consulting and MH works for Square Toiletries Limited. The remaining authors declare that the research was conducted in the absence of any commercial or financial relationships that could be construed as a potential conflict of interest.
